# Modified dose difference method for comparing dose distributions

**DOI:** 10.1120/jacmp.v13i4.3970

**Published:** 2012-07-05

Dear Editor‐in‐Chief,

I would like to comment on the article by Jino Bak, Jin Hwa Choi, Jae‐Sung Kim, and Suk Won Park, “Modified dose difference method for comparing dose distributions,” *J. Appl. Clin. Med. Phys. 2012; 13(2): 73–80.*

Something almost identical to their method was in fact first described by Bakai et al.^(^ ^1^ ^)^ who called their dimensionless index χ. While it is true that Bak et al. refer to this work in Reference 13, the authors do so in a list of variations on the γ index. However, γ indices involve a search over dose and spatial coordinates, while χ is a gradient‐dependent function like theirs, and I do not think that they properly acknowledge the similarity. The difference between their “modified dose” index and χ lies in the dimensionless parameter β, described in their Eq. (1) as:

(1) $$\beta =|\nabla D|\cdot (r_{\text{cri}}/D_{\text{cri}})$$

where $r_{\text{cri}}$ and $D_{\text{cri}}$ are distance‐to‐agreement and dose difference criteria (my symbols), and ▽D is the gradient of the reference dose distribution. Where the authors define a modified dose difference,

(2) $$\delta^{\left(M\right)}D=\delta D/(1+\beta ),$$

Bakai et al. would define the index χ as

(3) $$\chi =\delta D/D_{\text{cri}}/{\left(1+\beta^{2}\right)}^{1/2}$$

for an actual dose difference δD at a point (x,y,z), when their Eq. (6) is cast in the above terminology. In addition, Bakai et al. provided a convincing theoretical basis for the ${\left(1+\beta^{2}\right)}^{1/2}$ factor, rather than (1+β), in terms of the minimum distance between two surfaces in (x,y,z,D) space when measured in appropriate units.

To be fair to the authors, Bakai et al. did not actually define a modified dose difference $\chi D_{\text{cri}}$, nor the weighting factor ${\left(1+\beta^{2}\right)}^{1/2}$ explicitly, so the similarity could easily have been missed. I realize it is a bit unseemly to reference one's own work, but I did make that definition myself in connection with evaluating 3D dose differences.^(^ ^2^ ^)^

Sincerely, Will Ansbacher
